# Impact of obesity and SARS-CoV-2 infection: implications for host defence - a living review

**DOI:** 10.1093/oxfimm/iqab001

**Published:** 2021-01-21

**Authors:** Felix Clemens Richter, Aljawharah Alrubayyi, Alicia Teijeira Crespo, The Oxford-Cardiff COVID-19 Literature Consortium, Sarah Hulin-Curtis

**Affiliations:** 1 Nuffield Department of Orthopaedics, Rheumatology and Musculoskeletal Sciences, Kennedy Institute of Rheumatology, University of Oxford, Oxford, UK; 2 Nuffield Department of Clinical Medicine, University of Oxford, Oxford, UK; 3 Division of Cancer and Genetics, Henry Wellcome Building, Cardiff University, Cardiff, UK; 4 Division of Infection and Immunity, Henry Wellcome Building, Cardiff University, Cardiff, UK

**Keywords:** obesity, SARS-CoV-2, influenza, virus, metabolism, immune response, COVID-19, inflammation

## Abstract

The role of obesity in the pathophysiology of respiratory virus infections has become particularly apparent during the current severe acute respiratory syndrome coronavirus 2 (SARS-CoV-2) pandemic, where obese patients are twice as likely to suffer from severe coronavirus disease 2019 (COVID-19) than healthy weight individuals. Obesity results in disruption of systemic lipid metabolism promoting a state of chronic low-grade inflammation. However, it remains unclear how these underlying metabolic and cellular processes promote severe SARS-CoV-2 infection. Emerging data in SARS-CoV-2 and Influenza A virus (IAV) infections show that viruses can further subvert the host’s altered lipid metabolism and exploit obesity-induced alterations in immune cell metabolism and function to promote chronic inflammation and viral propagation. In this review, we outline the systemic metabolic and immune alterations underlying obesity and discuss how these baseline alterations impact the immune response and disease pathophysiology. A better understanding of the immunometabolic landscape of obese patients may aid better therapies and future vaccine design.

## INTRODUCTION

Obesity has emerged as an unexpected feature of severe acute respiratory syndrome coronavirus 2 (SARS-CoV-2) causing coronavirus disease 2019 (COVID-19) [1]. The SARS-CoV-2 pandemic has highlighted the global health crisis that is obesity [2] representing a risk factor independent of other co-morbidities [3]. Evidence of a link between obesity and infectious respiratory disease first became apparent after the 2009 influenza A pandemic (H1N1pdm). Obesity accounted for 12% of total deaths from H1N1pdm [4–7]. Early COVID-19 data emerging from China did not report body mass index (BMI), however, a study in Seattle showed that for critically ill patients, 85% were obese requiring mechanical ventilation and 62% of them died [8]. Two retrospective studies in New York City at the epicentre of the outbreak found COVID-19 patients aged <60 years with a BMI of 30–34 were 2.0 and 1.8 times more likely to suffer severe disease requiring critical care and a BMI ≥ 35 were 2.2 times more likely [9]. A BMI > 40 is a strong predictor of hospitalization (OR 6.2, 95% CI 4.2–9.3) [10]. In a French centre, 47.6% of severe COVID-19 patients were obese (BMI > 30) and 28.2% were severely obese (BMI > 35) with a total of 68.6% of patients requiring invasive mechanical ventilation, particularly males [3]. In the UK, several prospective studies based on UK biobank data sets revealed detrimental effects of obesity on COVID-19 susceptibility and disease severity, even after adjustment for other co-morbidities such as age, lifestyle and chronic diseases [11, 12]. Interestingly, Ho *et al*. demonstrated that increased inflammation which is associated with increased adiposity may increase susceptibility for severe COVID-19 in obese individuals [12]. Data from 265 patients hospitalized with severe COVID-19 disease, revealed a significant inverse correlation between age and BMI. Younger individuals (<50 years) who were admitted to intensive care units for COVID-19 were more likely to be obese compared with older individuals [13]. These studies highlight the risk of obesity in this generally considered low-risk age group [9, 13].

In this review, we will discuss how obesity impacts viral shedding, propagation, host metabolism, inflammation and immunity. Furthermore, we highlight ongoing therapeutic approaches to improve COVID-19 patient survival in the obese.

### Viral shedding

Obesity may play a role in prolonged viral shedding of infected individuals, potentially increasing transmission within the population. Viral shedding of IAV in obese adults can last 42% longer than lean individuals and in asymptomatic cases 104% longer [14], data confirmed in diet-induced obesity (DIO) mouse models [15]. Similarly, obese patients with SARS-CoV-2 required longer hospitalization stays and time to test negatively by pharyngeal swab compared to non-obese patients [16]. This indicates that obese individuals may require longer time to overcome viral infections and may promote viral spread in the population.

### Viral receptors

Obesity may contribute to viral propagation by promoting the expression of the SARS-CoV-2 cell entry receptor angiotensin-converting enzyme 2 (ACE2) on a variety of cell types including bronchial epithelial cells [17, 18]. ACE2 is also expressed on adipocytes [19, 20]. Importantly, obesity may upregulate ACE2 [21], possibly under the control of sterol regulatory element-binding protein 1 (SREBP1), a transcription factor which has an important role in lipogenesis, adipogenesis and cholesterol homeostasis to prevent lipotoxicity. Mouse studies show that ACE2 mRNA expression remained elevated in high-fat-fed mice compared with low-fat-fed mice demonstrating that adipocytes express ACE2 and is nutritionally regulated by high-fat feeding [22]. These findings suggest that obese people should be considered an at-risk population as ACE2 expression might be higher than in non-obese people. Indeed, ACE inhibitors (ACEi) represent good candidates for COVID-19 treatment [23–27] with telmisartan (NCT04355936) completing Phase IV and losartan (NCT04311177; NCT04312009) and captopril (NCT04355429) in Phase II Clinical Trials. All of these drug candidates are effective therapies for high blood pressure and heart failure, which are comorbidities more common in obese individuals. Telmisartan and losartan are angiotensin II receptor type 1 (AT1) antagonists and thus selectively block the binding of angiotensin II to the AT1 receptor [28]. This competitive replacement of endogenous angiotensin II will lower the pro-inflammatory and vascular effects by receptor activation and therefore may prevent hyperinflammation in COVID-19 patients. In contrast, captopril directly inhibits the enzymatic function of ACE preventing the conversion of angiotensin I to angiotensin II thus also increasing activation of the renin–angiotensin–aldosterone system (RAAS) [28]. The usage of ACEi has sparked debate about its safety since blocking RAAS can increase expression of ACE2 on the cell surface and thus could enhance viral uptake. On the contrary, the receptor is associated with tissue-protective, anti-inflammatory pathways and vasodilation [29] and could therefore reduce inflammation and cardiovascular insults caused by COVID-19. Early prospective cohort studies from more than 8 million participants in the UK suggest a reduced risk of COVID-19, when individuals were taking ACEi [30]. However, only completion of clinical trials will reveal whether ACEi can reduce COVID-19 risk and mortality associated with cardiovascular events.

### Viral impact on cellular lipid metabolism

Upon viral infection, SARS-CoV-2 hijacks cellular metabolism for replication and production of new virions. SARS-CoV-2 infection in monocytes leads to an accumulation of intracellular lipids and an increase of proteins involved in lipid metabolism such as the fatty acid transporter CD36 and lipogenic transcription factors such as the peroxisome proliferator-activated receptor γ (PPARγ) and SREBP-1 [31]. Furthermore, SARS-CoV-2 may use lipid droplets as replication sites. Pharmacological inhibition of diacylglycerol O-acyltransferase 1 (DGAT1), a key enzyme for triacylglyceride synthesis and lipid droplet formation, prevents viral replication [31]. Similarly, IAV infection increases the extracellular uptake of palmitic acid promoting viral replication, whereas blocking CD36-dependent palmitate uptake using sulfo-N-succinimidyl oleate prevents enhanced viral replication [32]. Circulating levels of palmitic acid are increased in obese individuals and can promote inflammation through binding to the pattern recognition receptors Toll-like receptor 2 and 4 [33]. In cells, palmitoylation of viral membrane proteins facilitate membrane fusion by SARS-CoV and some IAV strains [34, 35]. Although the mechanism has not been elucidated, an increase of palmitoylation of some proteins in obese mice suggests an alteration in this pathway [36, 37].

Besides palmitoylation, enveloped viruses up-regulate cholesterol metabolism [38]. Cholesterol is required for the formation of lipid rafts and is thought to assist viral spreading by serving as an assembly point at the plasma membrane and to promote efficient viral cell entry [39]. Due to the central role of cholesterol metabolism in viral replication, the lipid-lowering therapy statins (HMG-CoA reductase inhibitors), have been investigated as a potential antiviral therapy. Two recent retrospective studies on the use of statins in SARS-CoV-2 survival revealed variable outcomes with one showing a 50% improved overall survival [40] whereas the other could not identify any survival advantage among the statin cohort [41].

Drugs targeting lipid metabolism present novel therapeutic tools for combating obesity-related viral infection through their central role in the viral infection cycle. Thus, statins and other lipid-lowering drugs represent a promising candidate for COVID-19 treatment in obese patients [42].

In the following section, we discuss how obesity changes the host’s metabolic and immunological state during viral infections and how this may provide a potent environment for respiratory viral infections such as SARS-CoV-2.

## THE OBESOGENIC HOST

### Systemic chronic inflammation

Enhanced adipose tissue deposition in the obese (particularly in the abdomen) not only creates alterations in abdominal pressure with inadequate lung function and reduced oxygen saturation [43], but also represents the ‘soil’ for chronic low-grade systemic inflammation by activated immune cells in response to over-nutrition [44–46]. Activation of the innate immune response in adipose tissues creates altered cytokine and adipokine profiles. Dysfunctional hypertrophic adipocytes promote a chronic inflammatory state by producing pro-inflammatory cytokines and accumulating pro-inflammatory ‘M1-like’ macrophages [47–49] thereby outnumbering anti-inflammatory ‘M2-type’ macrophages that maintain normal tissue homeostasis [50]. This underlying chronic inflammation impairs innate and adaptive immune responses to pathogens [51] therefore leaving the obesogenic host vulnerable to viral persistence, secondary infection and vaccine failure [52].

Obese individuals infected for example with IAV show delayed and attenuated innate and adaptive immune response with reduced numbers of activated and functional memory CD4^+^ and CD8^+^ T cells, leading to increased viral spread with prolonged infection, lung damage and susceptibility to secondary bacterial infections [53]. Titers of influenza virus-specific antibodies increasingly decline at 1-year post-vaccination in obese vaccinees, increasing their 2-fold risk of severe/critical influenza infection and increased hospital admissions compared to their healthy weight counterparts [4].

Impaired B and T cell responses are a hallmark feature of severe COVID-19 disease [54]. Low naïve CD4^+^ and CD8^+^ T cells (lymphopenia) and CD4^+^ T cells with Th17 and Th22 phenotypes represent impaired adaptive immunity and a pro-inflammatory phenotype [55, 56]. Other studies show increased numbers of CD4^+^ T cells, reduced cytotoxic CD8^+^ T cells [57] combined with reduced regulatory T cells [58]. Activated T cells infiltrate adipose tissue possibly in response to antigens developed through high-fat feeding [59], thus contributing to local tissue inflammation. Coupled with T helper cells producing inflammatory cytokines, including IL-6, IL-10 and TNF-α in both obesity and COVID-19 patients, these factors influence disease progression [3]. Obesity promotes increased effector and memory T cell populations but reduced T cell receptor diversity compared with lean normal chow-fed mice [60]. Thus, obesity may impact the ability of circulating T cells to respond to a disparate pool of antigens, leaving the obese individual susceptible to infection. Adipose tissues represent an important site for many adaptive immune cells, in particular, to provide a metabolic niche for memory T cell development and maintenance supporting a protective response to infection [61]. During reinfection, antigen-specific memory T cells from adipose tissue show stronger activation and increased lipid uptake compared to memory T cells from lymphoid organs. Interestingly, obesity increases memory T cell numbers in white adipose tissue in a DIO mouse model. While able to clear lymphocytic choriomeningitis virus (LCMV) during primary infection, restimulation of cross-reactive memory T cells during a heterologous challenge promotes a pathological, lethal effect in obese mice. Thus, pathogenesis occurs when increased numbers of memory T cells kill virus-infected white adipose tissue and release IFNγ and TNF cytokines [62].

In contrast, little is known about the effects of obesity on B cell function during infection. Adipose tissue-derived B cells from lean animals protect against peritoneal antigens via T cell-dependent class switching and hypermutation [63]. Obesity increases the number of B cells in visceral adipose tissue and produces autoreactive immunoglobulins [64]. Two subpopulations of B cells (BRegs and B-1a cells) regulate glucose metabolism through secretion of the anti-inflammatory cytokine IL-10. The protective effects of B-1a cells through IL-10 secretion support normal insulin regulation. In diabetic patients and obese mice, the function of B-1a cells is impaired, promoting chronic, low-grade inflammation and insulin resistance [65]. Furthermore, B-1 cells have been shown to promote protection to influenza infections by producing cross-reactive natural IgM [66]. However, it remains unclear if antibody-secreting cells accumulate in adipose tissues upon primary infection or vaccination and whether they contribute to the secondary response during reinfection. The formation of an adaptive immune memory to SARS-CoV-2 may be crucial to identify the best vaccination strategy for the obese population that is so much more at risk of severe disease course.

### Dyslipidaemia

Lipid homeostasis is predominantly regulated by adipose tissues and the liver in order to provide cells with essential lipids for biosynthesis and energy metabolism. Many lipids, including cholesterol, are transported in lipoprotein complexes in blood to avoid lipotoxicity. These are crucial for immune and stromal cell function. For instance, macrophages rely on high-density lipoproteins (HDLs) to control cholesterol efflux [67]. Obese individuals have lower levels of high-density lipoprotein cholesterol (HDL-C) that leads to an accumulation of cholesterol in cells which can promote inflammation and viral replication while decreased total circulating cholesterol levels limit T cell expansion [68]. A common feature of viral infections is an imbalance in systemic lipid metabolism often leading to a significant reduction in circulating HDL-C levels. In line with this, severe to critical SARS-CoV-2 patients exhibit reduced HDL-C levels which are inversely correlating with inflammation markers such as C-reactive protein [69, 70]. Knowing that dysregulation of HDL-C levels occurs frequently in critical viral infections, treatment of this imbalance by administration of an Apo-A1 mimetic, the main lipoprotein in HDL-C complexes, significantly reduced influenza-induced lung damage in a murine model [71]. Taken together, this would suggest that decreased circulating HDL-C due to obesity may raise the risk of increased disease severity in viral infections.

Besides cholesterol, other lipid species can directly impact on immune function such as derivates of arachidonic acid. This poly-unsaturated fatty acid is transformed in innate immune cells to lipid mediators known to promote inflammation such as prostaglandins. COVID-19 patients show a gradual decrease in the precursor arachidonate with increasing disease severity [72]. This may indicate that SARS-CoV-2 can propagate more efficiently. In line with this, arachidonic acid supplementation can limit Middle East Respiratory Syndrome (MERS) virus production [73]. Other lipid mediators derived from eicosanoid acid and docosanoic acid are also dysregulated in SARS-CoV-2 patients and show an overall increase with disease severity, with some pro-resolving lipid mediators showing decreased presence in severe patients [74]. This is in line with observations of highest cyclooxygenase activity in moderate compared to severe patient groups. Furthermore, the activity of the arachidonate 5-lipoxygenase (ALOX5), a key enzyme in the production of arachidonic-derived mediators exhibits increased expression in obese individuals and severe COVID-19 patients. ALOX5 is predominantly expressed in monocytes and macrophages of SARS-CoV-2-infected patients and may contribute to inflammation through increased leukotrienes which can promote pro-inflammatory adipokine production [75–77]. Similar changes have been observed in influenza patients [78]. Based on these studies, it is likely that the underlying obesity-associated lipid imbalance is further worsened by SARS-CoV-2 promoting development of severe disease pathology, impairing resolution and hence prolonged inflammation.

### Oxidative stress and mitochondrial dysfunction

Mitochondria play a key role in cellular metabolism including tricarboxylic acid cycle, decarboxylation of fatty acids or β-oxidation to generate and sustain adenosine triphosphate (ATP) levels for cellular energy demands, while also being the main producer of reactive oxygen species (ROS). Accumulating evidence links the accelerated progression of COVID-19 to increased levels of inflammation leading to increased ROS production and cellular oxidative stress which in turns can trigger mitochondrial dysfunction [79]. Indeed, oxidative stress was described as a main player in COVID-19 pathogenesis [80] and oxidative stress-associated genes were enriched in bronchoalveolar lavage fluid of severe COVID-19 patients [81]. This was demonstrated in SARS-CoV-2-infected monocytes, which play a key role in SARS-CoV-2-driven inflammation [81], displaying higher mitochondrial ROS production and reduced mitochondrial oxidative metabolism including a reduction in the spare respiratory capacity, a critical complement of mitochondrial bioenergetics that can be utilized during increased energy demands [82]. In agreement with these findings, mitochondrial antioxidant treatment leads to inhibition of SARS-CoV-2 replication and prevents upregulation of inflammatory cytokines [83]. This suggests dysregulated mitochondrial function of monocytes could contribute to SARS-CoV-2 pathogenesis.

Obesity causes profound metabolic alterations leading to mitochondrial dysfunction [84–86] which can contribute to obesity-related COVID-19 pathogenesis. Imbalance of nutrient intake, high free fatty acids concentration and hyperglycaemia result in increased ROS production and adipocyte mitochondrial dysfunction [87]. Consistent with these findings, adipocytes in obese hosts display a reduced mitochondrial oxidative capacity and biogenesis [85, 86] and a downregulation of fatty acid oxidation (FAO) and the tricarboxylic acid cycle pathways which inversely correlate with low-grade inflammation [85, 88]. These obesity-associated host factors could serve as dual roles in the metabolism of virus-infected cells. First, these factors may ‘fuel the fire’ of viral-induced metabolic reprogramming and mitochondrial alterations. Second, obesity-associated mitochondrial defects can contribute to modulating antiviral immune responses [53, 79]. For example, lipid accumulation in natural killer (NK) cells leads to impaired NK cellular metabolism, including mitochondria respiration, and trafficking of the cytotoxic machinery, causing a complete ‘paralysis’ of their cytotoxicity [89]. In addition to NK cells, accumulation of lipids in dendritic cells (DCs) results in reduced capacity in processing antigens, leading to a defect in stimulating allogeneic T cells [90]. Type-17 mucosal-associated invariant T (MAIT) cells which have been reported to increase in obese hosts [91] and linked to the pathogenesis of chronic infections [92], displayed altered mitochondrial metabolism in obesity [92]. Interestingly, these subsets have been described as the prominent IL-17-producing cells in the airways of COVID-19 patients [93].

### T cell metabolism

In the context of acute viral infection, increased energy demand for effector T cell function is derived from a variety of fuel sources. Shifting from oxidative phosphorylation to glycolysis and production of ATP provides the fuel for increased metabolic demands. The conversion of effector T cells to long-lived memory T cells requires a switch back to oxidative metabolism with fatty acids as the fuel source [94, 95]. Additionally, the maintenance of tissue-resident memory CD8^+^ T cells depends on the uptake of exogenous free fatty acids [96]. Although T cells respond to antigens by altering their metabolic state in this way, it is not clear how systemic metabolic conditions affect T cell function. Saturated fatty acid-induced metabolic alteration leads to a preferential migration of effector T cells to inflammatory sites, contributing to low-grade systemic inflammation observed in obese individuals [97]. Treatment with palmitic acid results in increased oxidative phosphorylation in naïve T cells promoting their differentiation into pro-inflammatory effector memory phenotype, suggesting a preferential usage of FAO during T cell activation in fat-rich environment promotes pro-inflammatory effector T cell differentiation [97]. Consistent with these findings, T cells isolated from the spleen of mice with DIO display altered mitochondrial phosphorylation and a preferential utilization of fatty acids as mitochondrial fuel. In COVID-19, T cells from patients with severe disease show an increased oxidative phosphorylation compared with mild or recovered groups, suggesting altered T cell metabolism in severe infection [98]. Furthermore, T cells from patients with progressed COVID-19 displayed an altered mitochondria morphology, increased mitochondrial mass and accumulation of ROS production. Interestingly, COVID-19 patients with pre-existing metabolic disorders such as obesity showed different capacities for nutrient uptake compared to uninfected controls [99]. In addition, obesity dysregulates glucose usage and FAO and storage [100, 101] and changes in T cell metabolism are associated with impaired T cell response to influenza. Even prior to influenza infection, obese mice had altered cellular metabolism characterized by T cells with increased oxidative phosphorylation and glycolysis [102]. After influenza infection, CD4^+^ and CD8^+^ T cells from obese mice significantly increased the oxidative phosphorylation to glycolysis ratio in comparison to lean mice [102]. Weight loss in obese mice reversed systemic hyper-insulinaemia and hyperglycaemia but failed to prevent infiltration of T cells into adipose tissue and did not reverse memory T cell dysfunction. These findings suggest that obesity and metabolic disturbance creates epigenetic reprogramming on T cell function. This has not been studied in SARS-CoV-2 patients but is likely to contribute as T cell responses from critically ill patients are significantly impaired.

## CONCLUSION

Obesity causes severe impairment of systemic lipid homeostasis due to calorie excess. Hypertrophic adipocytes create a state of systemic lipid imbalance and low-grade inflammation. This change in the host’s metabolic and inflammatory status renders immune cells dysfunctional by substantially altering mitochondrial structure and function. Consequently, immune cell metabolism shifts away from oxidative phosphorylation towards more pro-inflammatory glycolytic pathways, exacerbating SARS-CoV-2-induced inflammatory processes. It remains to be seen if obese patients can mount an effective memory B and T cell response to SARS-CoV-2 infection since dysfunctional adipose tissues in obese individuals may impair the necessary immunometabolic switch of memory cells towards FAO and consequently alter their reactivation potential. Previous studies examining influenza infections show that obesity impairs memory responses leaving the host more susceptible to reinfection. It remains to be seen whether obese patients are more likely to experience reinfection with SARS-CoV-2 over the coming years. Dyslipidemia may further promote viral replication through increasing ACE2 expression on adipocytes and epithelial cells.

Although global health measures to reduce obesity may represent a long-term solution to reduce obesity-driven impairments in immune response to viral infections [103], patients are in desperate need of new and effective treatments and vaccines to reduce disease severity and mortality. Drugs targeting host and or viral lipid metabolism are currently under investigation and statins have shown early promising results in promoting overall patient survival in retrospective studies [40]. However, it remains unclear whether the effects are mediated through their impact on the host’s lipid metabolism or through direct anti-inflammatory effects or both.

Future studies elucidating the metabolic and immunologic mechanisms of obesity and risk of SARS-CoV-2 infection and severe disease are warranted. Understanding these pathways at the molecular level may enable development of targeted therapeutic approaches and aid future vaccine design for the obesogenic host.

**Box 1:** Why does obesity matter during COVID-19?

Several studies underline the significant changes in lipid metabolism observed during viral infections, some of which are reminiscent of changes observed in obese uninfected individuals. It is clear that obese individuals are 2 times more likely to experience severe COVID-19 progression. This shows striking parallels to heightened risk of influenza-related hospitalization and disease severity. Obesity fundamentally alters the host’s metabolism and promotes obesity-associated inflammation, which has been linked in the context of influenza infections to increased viral replication and disease severity. Thus, obese individuals represent a more susceptible population group that requires particular attention in disease prevention and treatment during this pandemic.

**Box 2:** What is the consensus on obesity and COVID-19?

Obesity in the UK has been increasing over the past decades. Today, ∼30% of the UK population is classified as obese. Obesity increases the risk of other chronic diseases and makes individuals more susceptible to viral infections like influenza and as observed now during the SARS-CoV-2 outbreak. The UK government has recently acknowledged the necessity of tackling this issue and started promoting long-term public health measures (see https://www.gov.uk/government/news/new-obesity-strategy-unveiled-as-country-urged-to-lose-weight-to-beat-coronavirus-covid-19-and-protect-the-nhs) [103] to decrease obesity in its population. However, it will take time until these measures show effects. It remains crucial to further continue broadening our understanding of the metabolic and immunological changes in obese individuals. Improving our understanding of how obesity promotes viral infections such as SARS-CoV-2 may lead to better therapies and vaccine designs in this patient group.
